# Supplementary material for: Can Bayesian phylogeography reconstruct migrations and expansions in linguistic evolution?
Source: R Soc Open Sci. 2021 Jan 13;8(1):201079. doi: 10.1098/rsos.201079 (PMC7890507; doi:10.1098/rsos.201079)
Supplement: Supporting Information: Can Bayesian phylogeography reconstruct migrations and expansions in linguistic evolution? [file rsos201079supp1.pdf]

Supporting Information: Can Bayesian phylogeography reconstruct migrations and expansions in linguistic evolution?

Nico Neureiter[a,b], Peter Ranacher[a,b], Rik van Gijn[c,d], Balthasar Bickel[a,c,e], and Robert Weibel[a,b,e]

[a]University Research Priority Program (URPP) Language and Space, University of Zurich
[b]Department of Geography, University of Zurich
[c]Department of Comparative Language Science, University of Zurich
[d]Leiden University Centre for Linguistics
[e]Center for the Interdisciplinary Study of Language Evolution (ISLE), University of Zurich

Contents

S1 Migration Simulation (supplementary to Section 3(a) i) **S1**
S1.1 Ancient languages (fossils) S1

S2 Expansion Simulation (supplementary to Section 3(a) ii) **S1**
S2.1 Growing areas S1
S2.2 Area splitting S1

S3 Reconstruction (supplementary to Section 3(b)) **S2**

S4 Evaluation (supplementary to Section 3(c)) **S2**
S4.1 HPD coverage S2

S5 Results for the RW and RDRW models **S3**

S6 Sensitivity analysis **S3**
S6.1 Sensitivity: Tree size S3
S6.2 Sensitivity: ExpSim area overlap S4

S7 Descriptive statistics **S4**
S7.1 Clade overlap S5
S7.2 Diversity-space dependence S7
S7.3 Tree imbalance S7
S7.4 Migrations, expansions, and Bantu S7

S8 Effect of missing first clades **S9**

This document provides supporting information for the main article “Can Bayesian phylogeography reconstruct migrations and expansions in human history?”. The first sections provide additional details on the simulations (S1, S2), phylogeographic reconstruction (Section S3) and evaluation (S4) in the main simulation study. Further we include results of additional experiments (S5), a sensitivity analysis (S6), a detailed explanation of the descriptive statistics used in the discussion of the main paper (S7) and a figure, illustrating the effect of missing first clades on the reconstruction (S8).

S1 Migration Simulation (supplementary to Section 3(a) i)

S1.1 Ancient languages (fossils)

In the MigSim scenario we simulated phylogenetic trees according to a birth-death process with birth rate $\lambda = 0.00115$ and death rate $\nu = 0.00023$. This yields trees with an average of $N = 100$ extant languages. The death rate allows us to simulate extinct languages, which we included in some of the experiments. In the Results section of the main article (4) we show three settings:

No historical data: only the extant languages are used for the reconstruction.

Sample age $\leq$ 500: include languages that went extinct in the last 500 years.

All sample ages: include all languages, extant or extinct.

We achieved these settings by filtering the extinct languages generated by the birth-death process to only include the specified ages. To ensure that the extinct languages have an observable effect, we defined a minimum fossil number of 10, i.e. we restarted the simulation when the filtering resulted in less than 10 fossils (except for the *“no historical data”* setting).

S2 Expansion Simulation (supplementary to Section 3(a) ii)

In contrast to the migration simulation, which is based on a well known random walk process, the expansion simulation is more novel and deserves some additional explanation. In what follows we give some details on the way in which the areas in ExpSim grow and split over time.

S2.1 Growing areas

At each step in the simulation, every simulated area has a probability p_{grow} of growing into a neighbouring cell. We implemented varying rates of spatial expansion by randomly drawing different values for p_{grow} in each language. We generated each p_{grow} from a uniform distribution over the interval $[0, 1]$. The growth is restricted to free cells, which are not occupied by any other language (we relax this constraint in the sensitivity analysis in S6.2). When no free neighbouring cells are left, the language area stops growing.

S2.2 Area splitting

The expansion simulation implements an area-dependent diversification process in the following way: As soon as the area of a language (in number of cells) grows beyond a randomly selected threshold, it will split into two daughter languages with disjoint areas. The splitting threshold is a parameter which is drawn uniformly from a range of 70 to 100 cells for each language (the effect of changing this range is shown in the sensitivity analysis S6.1). The split size of 70 to 100 cells along with the geographic constraints results in extant language with an area of approximately 50 cells on average, equivalent to approximately 1% of the whole simulated area (this was stable across the different cone angle settings). In order to run the phylogeographic analyses, we eventually represent the simulated language areas by their centre of mass (the tested methods require a point location).

When a language splits, the cells of the area are divided along a straight line and each daughter language inherits the cells on one side of this line. To ensure that the daughter languages still comprise reasonably compact areas, we split the previous area along the axis of maximum variance (illustrated in Figure S1).

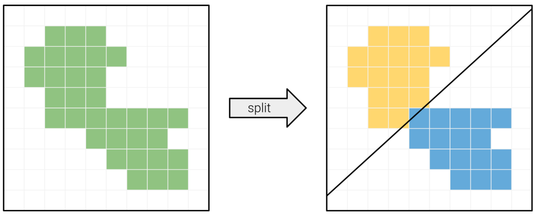

Figure S1: Areas in ExpSim are split along straight lines.

S3 Reconstruction (supplementary to Section 3(b))

The phylogeographic reconstructions were carried out in BEAST 1.10.4. As described in Section 3(b), we fixed the phylogenetic tree in the analyses and were interested in the reconstruction of the root location. Fixing the input tree makes the analysis simpler, since we do not need to specify a tree prior, substitution model or clock model for linguistic data. The only relevant parameters are the ones defining the spatial diffusion model and the settings for the MCMC inference.

The RRW model is defined by the precision matrix of the diffusion process and by the branch specific diffusion rate. For the precision matrix, we used a Wishart prior with two degrees of freedom and the scale matrix set to be the identity matrix. The diffusion rates follow a log-normal distribution with mean 1. The standard deviation is estimated and has an exponential prior with mean 0.333. The directional random walk models (CDRW and RDRW) additionally required us to estimate the drift parameter. As a prior on the drift parameter we used a normal distribution with mean 0.0 and standard deviation 0.1. Furthermore, in the RDRW model the number of trend changes follows a Poisson distribution with mean 0.693.

BEAST uses Markov chain Monte Carlo (MCMC) to generate posterior samples from the model. We ran the MCMC for 10^6 iterations and discarded the first 10^5 iterations as burn-in. This might seem low compared to other phylogenetic analyses, but fixing the phylogenetic tree reduces the sampling complexity significantly. This was also crucial in allowing us to explore the range of different scenarios presented in this study.

These and other settings are defined in the XML templates and experiment scripts on our GitHub repository (https://github.com/NicoNeureiter/drifting_into_nowhere/).

S4 Evaluation (supplementary to Section 3(c))

S4.1 HPD coverage

To evaluate whether the uncertainty shown in the posterior distribution of the phylogeographic analysis reflects the reconstruction errors observed in our experiments, we reported the 80% and 95% HPD coverage. That is, we used TreeAnnotator (part of the BEAST toolbox [1]) to compute spatial polygons, which contain 80% (or 95%, respectively) of the probability mass with the highest density, according to a spatial kernel density estimation (KDE) on the posterior samples. The coverage of these HPD polygons is the fraction of simulation runs in which polygons contain the simulated homeland location. If the model perfectly reflects the uncertainty in the reconstruction, the 80% (or 95%) HPD polygon should cover the true homeland in 80% (or 95%) of the runs, yielding a coverage of 0.8 (or 0.9). A higher coverage indicates an underconfident model, i.e. the posterior shows too much uncertainty, while a lower coverage indicates an overconfident model, i.e. the posterior shows too little of the true uncertainty.

S5 Results for the RW and RDRW models

In the main paper we have introduced four phylogeographic models: the random walk (RW; also Brownian motion), relaxed random walk (RRW), constant directional random walk (CDRW) and relaxed directional random walk (RDRW) models. We performed simulation studies with all of these, but only presented the results for RRW and CDRW model in Section 4. The reason in the case of the RW model is that the assumption of constant rates is oversimplifying, the model is rarely used in phylogeographic studies and unsurprisingly it yielded inferior results to the other models. In the case of the RDRW model, the results are practically the same as in the CDRW model. This is also unsurprising, since the directional trends in our simulations do not change over time, as modelled by the RDRW model. We show the results for all four models in Figures S6 and S7.

In Figures S6 and S7, as well as Figures 4 and 5 in the main article, we presented all performance measures in relation to $\hat{\mu}$, the **observed trend**. The observed trend (described in (c)) is a statistic that can be computed on either simulation scenario, independent of the spatial process, but it is not a parameter of these processes. The parameters controlling the trend are different in the MigSim and ExpSim scenario: in the former it is μ, the total trend of the random walk, and in the latter it is α, the sector angle of the geographic constraints. This makes it hard to compare results between simulations, which was the motivation for using the observed trend. Nevertheless, we also show the results directly in terms of the controlled parameters in Figures S8 and S9. For the MigSim results, the change from $\hat{\mu}$ to μ is hardly noticeable. When looking at the ExpSim results, it is important to keep in mind that lower sector angles lead to stronger directional trends (i.e. the x-axis is flipped).

S6 Sensitivity analysis

In the main article we have presented two simulation scenarios: migration simulations (MigSim) and expansion simulations (ExpSim). These two scenarios lead to very different performance in the reconstruction of the simulated histories, which is a core part of our findings. To ensure that these findings are really stable properties of the two simulation scenarios and do not depend on the specific settings of some parameters, we performed a sensitivity analysis, which we present here. In the first analysis we will test the effect of the size of the simulated phylogenetic trees (S6.1). In the second analysis we explore the effect of relaxing the assumption that languages can not overlap in the expansion simulations (S6.2).

S6.1 Sensitivity: Tree size

In the MigSim scenario it is fairly straightforward to vary the tree size, while keeping other parameters fixed: We simply set the birth-rate to $\lambda \in \{0.0009, 0.0012, 0.00143\}$ (and the death-rate accordingly to $\nu \in \{0.00017, 0.00023, 0.00029\}$), leading to an expected tree size of 50, 100 and 300, respectively. The results of these experiments are shown in Figure S2. We can see that the results are, indeed, very robust to variations of the tree size.

For the expansion simulations there is no analytical formula to compute the expected tree size from the parameters, thus adjusting the tree size requires some tuning. As in the MigSim scenario, we change the diversification process, in this case by adjusting the size at which languages split. In the default case the split size was randomly chosen between 70 and 100, leading to a tree size of about $N = 100$. We varied this split size range between $\{140, ..., 200\}$ and $\{25, ..., 33\}$ to obtain trees with about 50 and 300 leaves, correspondingly. As we can see in Figure S3, changing this parameter has a more notable effect here. Firstly, the simulations show a slightly lower observed trend with smaller trees (i.e. when we increase the split size of the areas). Secondly, the reconstruction error increases and the HPD coverage decreases for small trees. Despite these differences our main findings hold: The reconstruction error (measured as RMSE and bias) levels off and is far away from the linear increase seen in the MigSim scenario. While the coverage does fall below the desired values of 80% and 95%, respectively, for small trees with certain trend values, in almost all settings the confidence regions seem to overestimate the uncertainty (i.e. we can trust the confidence regions provided by the reconstruction).

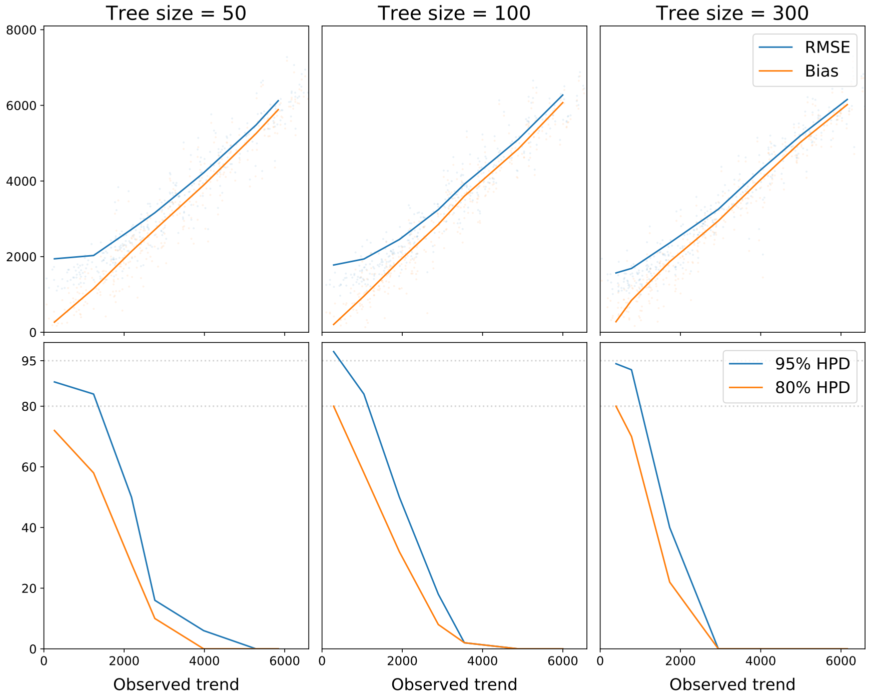

Figure S2: Results for the MigSim scenario, with varying tree size. The top row depicts the bias and RMSE of the reconstruction, the bottom row the 80% and 95% HPD coverage.

S6.2 Sensitivity: ExpSim area overlap

In human history multilingualism and coexistence of different populations in the same place are the rule rather than the exception. In contrast to this, the original version of the ExpSim simulation only allows language areas to grow into free cells (not inhabited by any other population). The motivation for this is the scenario of populations expanding into new areas, where their languages where not spoken before, e.g. in search of available land to grow their crop. Still, we want to be sure that our findings do not break down if this hard constraint is relaxed. To do this we ran an analysis on a modified simulation where overlaps between areas are allowed within two constraints:

1. The number of languages per cell must not exceed 3. Without a limit on the number of languages per cell, the languages would diversify exponentially within an area before they even get to expand into new areas (which happens at a quadratic rate). Simulating a significant geographic expansion without this constraint would thus lead to unrealistic and computationally problematic clusters of many languages in the same area.

2. Languages are more likely to expand into free cells than occupied cells. The probability of growing into an occupied cell is reduced by a factor of ρ_{overlap} which we vary between 0 and 1, where 0 corresponds to the standard setting with disjoint areas and 1 corresponds to a setting where the occupancy of a cell does not matter (up to the limit of the first constraint, i.e. 3 languages).

As can be seen in Figure S4 the reconstruction bias increases when we allow language areas to grow into each other, but even in the extreme scenario of $\rho_{\text{overlap}} = 1$, where previous (up to three) languages are completely ignored, the error levels off. That is, our findings regarding the ExpSim scenario indeed withstand the relaxation of the overlap constraint.

S7 Descriptive statistics

The two simulation scenarios for migrations (MigSim) and expansions (ExpSim), show very different patterns, not only in the spatial distribution of the languages, but also in the tree shape. We visualise these differences in Figure 6 of the main paper.

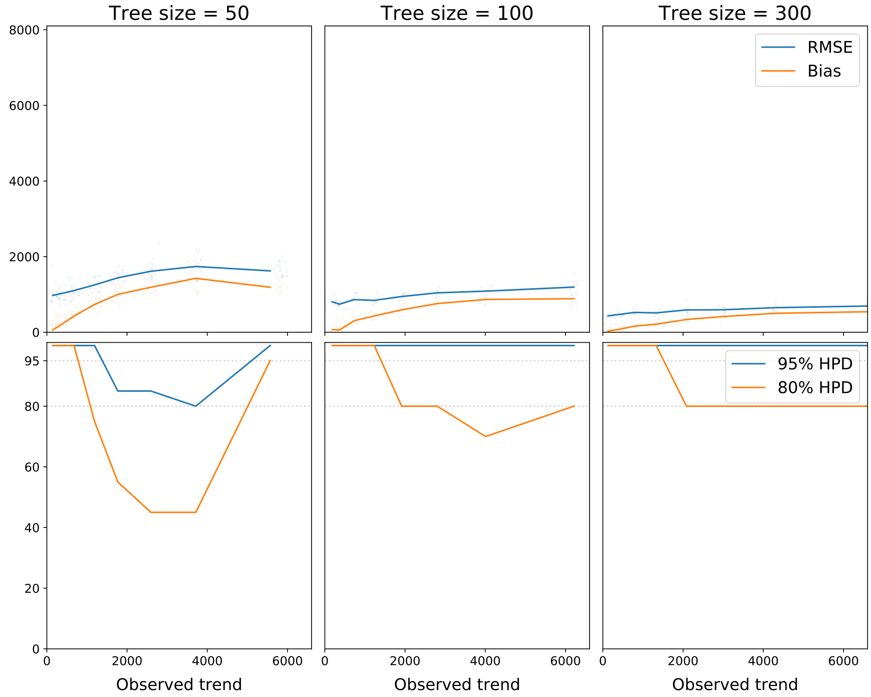

Figure S3: Results for the ExpSim scenario, with varying tree size. The top row shows the bias and RMSE of the reconstruction, the bottom row the 80% and 95% HPD coverage.

In the MigSim scenario, the diversification and the movement processes are independent, and under common tree priors we would expect a relatively balanced tree topology. The movement follows a random walk along this tree with a bias in one direction, as visualised in the middle row of Figure 6. In the expansion scenario on the other hand, the diversification and spatial expansion are strongly dependent. This leads to different spatial and phylogenetic patterns from random walks (right column of Figure 6).

In the ExpSim scenario there is no intrinsic bias for languages to move in one direction or to move at all. Actually, every language tends to stay sedentary and it is only the diversification and spatial expansion that is forced to progress in one direction due to geographic constraints (and closer areas being occupied by previous sedentary languages). As a result the region of the homeland will always be populated, mostly with languages that stopped to diversify and to migrate a long time ago (compared to the languages at the frontier of the expansion). In Figure 6 (right column) we illustrate what such an expansion typically looks like, with an unbalanced tree topology (top), where languages split off one after another. The languages splitting off earlier remain closer to the homeland, while the expansion spreads away, causing the directional trend (middle row of Fig. 6).

The observations made above regarding these different processes can be expressed by three measurable properties, which we describe below. The spatial dimensions of these properties are based on point locations of languages. In the ExpSim scenario we take the centroid of an area as the point location.

S7.1 Clade overlap

The ExpSim scenario enforces disjoint language areas, which cannot cross each other in the course of the expansion. This means that the clades of the phylogeny will cover non-overlapping geographic areas. In the MigSim scenario on the other hand, the languages can move freely and independently in space, leading to significant overlap between the clades. To quantify the concept of clade overlap in a measurable score, we need to do two things:

1. Define the clades to be compared.
2. Measure the overlap between the clades.

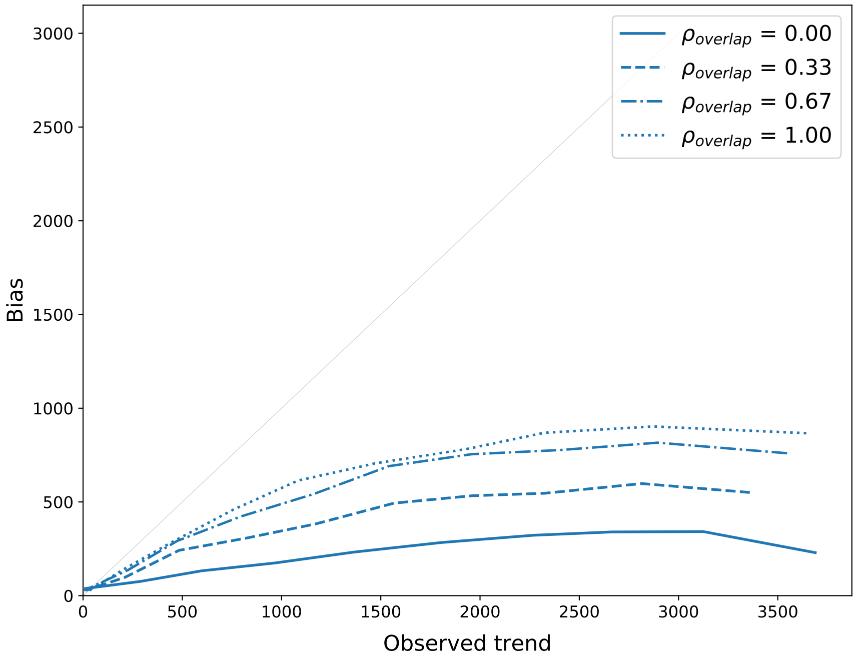

Figure S4: The reconstruction bias (see Section 3(c)) in the ExpSim scenario with varying overlap coefficient ρ_{overlap}.

Clades For clades to be comparable we fix a tree height h and define clades as all the sub-trees starting in lineages at that height. We set h to half the total tree height. The rationale here is that we want enough clades to compare, but also clades of a sufficient size to measure a meaningful overlap (i.e. balancing out the number of clades and clade size).

Join count statistic To quantify the overlap between clades, we follow a simple observation: If clades overlap, nodes will have spatial neighbours from different clades, if they do not overlap, the spatial neighbours will mostly be from the same clade. We use the Delaunay triangulation to define spatial neighbourhood (so-called 'natural neighbours') and measure the fraction of neighbours in the same clade according to the *join count statistic*. More specifically, we count all edges (i, j) in the Delaunay graph G where both nodes are in a certain clade $C \subseteq V(G)$ and denote the result by

$$n_{\text{bb}}(G, C) := |\{(i, j) \in G | i \in C \wedge j \in C\}|.$$

Clearly, n_{bb} strongly depends on the size of the clade (the larger C, the higher we expect $n_{\text{bb}}(G, C)$ to be). Thus, we define a normalised *compactness* score α_C, such that $\alpha_C = 0$ corresponds to a scenario where clades are completely randomly mixed in space (i.e. no spatial auto-correlation of clades), while $\alpha_C = 1$ corresponds to a scenario of maximal connectedness within a clade. We perform this normalisation by computing the expected join count in the former and the latter scenario (denoted by n_0 and n_1). We can compute n_0 by shuffling the clade labels of all locations and compute the n_{bb} score based on the shuffled labels:

$$n_0(G, C) = \mathbf{E}_\pi[|\{(i, j) \in G \mid \pi(i) \in C \wedge \pi(j) \in C\}|]$$

Where the expectation is taken over permutations π on the vertices of G.

As an estimate of n_1, we take Euler's formula, which defines an upper bound on the edges in a planar graph:

$$|E(G)| \leq 3\,|V(G)| - 6$$

Since the Delaunay triangulation is planar and a clade cannot have more internal edges than a maximal planar graph of that size, this directly translates to an upper bound on n_{bb}:

$$n_{\text{bb}}(G, C) \leq 3\,|C| - 6$$

We then normalize the join counts according to a min-max scaling for these two values:

$$\alpha_C = \frac{n_{\text{bb}}(G, C) - n_0}{n_1 - n_0}$$

Not that n_0 is not really a minimum: in cases of systematically intertwined clades n_{bb} may even be lower than in the purely random case. This would result in $\alpha_C < 0$. However, we neither expected nor observed negative values for α_C in our experiments.

As a last modification to the score, we want to bring the measure in line with our chosen terminology of clade overlap. α_C measures the compactness of a clade, which is large if the overlap is low. Thus, we invert the score to obtain the overlap score used in our experiments:

$$\text{overlap} = 1 - \alpha_C$$

S7.2 Diversity-space dependence

In the ExpSim scenario we model diversification as a result of spatial expansion: speakers in larger areas are less in contact, which leads to language divergence and eventually a split into two distinct languages. This implies that a clade of languages that is expanding faster will also diversify more (i.e. result in more languages). This effect is observable in a correlation between the rate of spatial expansion of a clade and the rate of diversification. Again, the MigSim scenario does not show such a pattern: In random walks (directed or not) the diversification and migration rates may vary, but there is no reason to expect these two to be correlated. We estimate the migration rate in a clade by the spatial variance divided by its age. Similarly, we define the log-diversification rate as the logarithm of the number of leaves divided by the clade age[1]. We measure the correlation between the migration and the log-diversification rate using the the Pearson correlation coefficient and denote this score by *diversity-space dependence*. The diversity-space dependence is expected to be close to 0 if movement and diversification are independent, and tends towards 1 if they are strongly correlated.

S7.3 Tree imbalance

Furthermore, we measure the imbalance of the phylogenetic tree. This captures a way in which the previous two observations about the ExpSim scenario – disjoint language areas and space-diversity dependence – interact: Due to disjoint language areas some clades will be restricted by other languages (and geographic constraints) in their spatial spread, while other clades at the frontier of the expansion can continue to grow into new areas. The space dependent diversification in turn means that these locked-in clades will not diversify as much and contain fewer languages. This effect manifests itself in an imbalanced tree. Tree imbalance has been studied extensively in the literature on phylogenetic tree shape [2] and researchers have proposed different scores to measure imbalance. We use the weighted imbalance score I introduced in [3]. This score has an expectation of 0.5 for an unbiased birth-death process and is lower for unexpectedly balanced and higher for unexpectedly imbalanced trees.

S7.4 Migrations, expansions, and Bantu

In Figure 7 of the main paper we show all three statistics for the MigSim scenario, the ExpSim scenario, and for the phylogeographic reconstruction of the Bantu expansion. We can see that the MigSim scenario produces migrations with high clade overlap, low diversity-space dependence and low tree imbalance compared to the ExpSim scenario. In fact, the diversity-dependence is (widely) distributed around 0 and the tree imbalance falls around 0.5, as expected, since the diversification dynamics and the movement dynamics are independent. In both cases the ExpSim scenario would clearly deviate from such a null hypothesis.

Further, we can see that the Bantu expansion strikingly follows the distribution of the ExpSim scenario. In the Bantu case we computed the statistics on every sample from the posterior distribution, yielding the plotted distribution of the statistics. Since the statistics from the Bantu

[1]We take the log-diversification rate because the number of leaves is growing exponentially with time, in contrast to the spatial variance, which grows linearly with time (in a random walk scenario).

expansion represent one historical scenario (rather than many simulated ones, as in MigSim and ExpSim), it is not surprising that the distributions are slightly narrower. The important observation is the clearly higher agreement with the ExpSim scenario, suggesting that the Bantu case study aligns with an expansion scenario.

We want to emphasise that we see these statistics as a first step towards a more detailed characterisation of historical migration processes and that any such statistic can only be part of a broader discussion of plausibility of reconstructions in a historical context.

S8 Effect of missing first clades

We also explored the effect of missing first clades, as it might occur due to language shifts, population shifts or uneven sampling. In the top panel of Figure S5 we render the reconstruction of the Bantu languages, based on the full sample from [4] (including an outgroup of Grassfields languages). The root – marked with a circle – is placed by the reconstruction in central/western Cameroon, close to the hypothesised homeland of Bantu languages. In the bottom panel we show the reconstruction based on a reduced sample, where we removed the outgroup and the first three clades. In line with our arguments about the importance of the first clades (Section 5(b)), we see that not including these clades has a severe effect on the reconstruction of the homeland. The result is also in line with the convexity property introduced in Section 5(a) of the main paper.

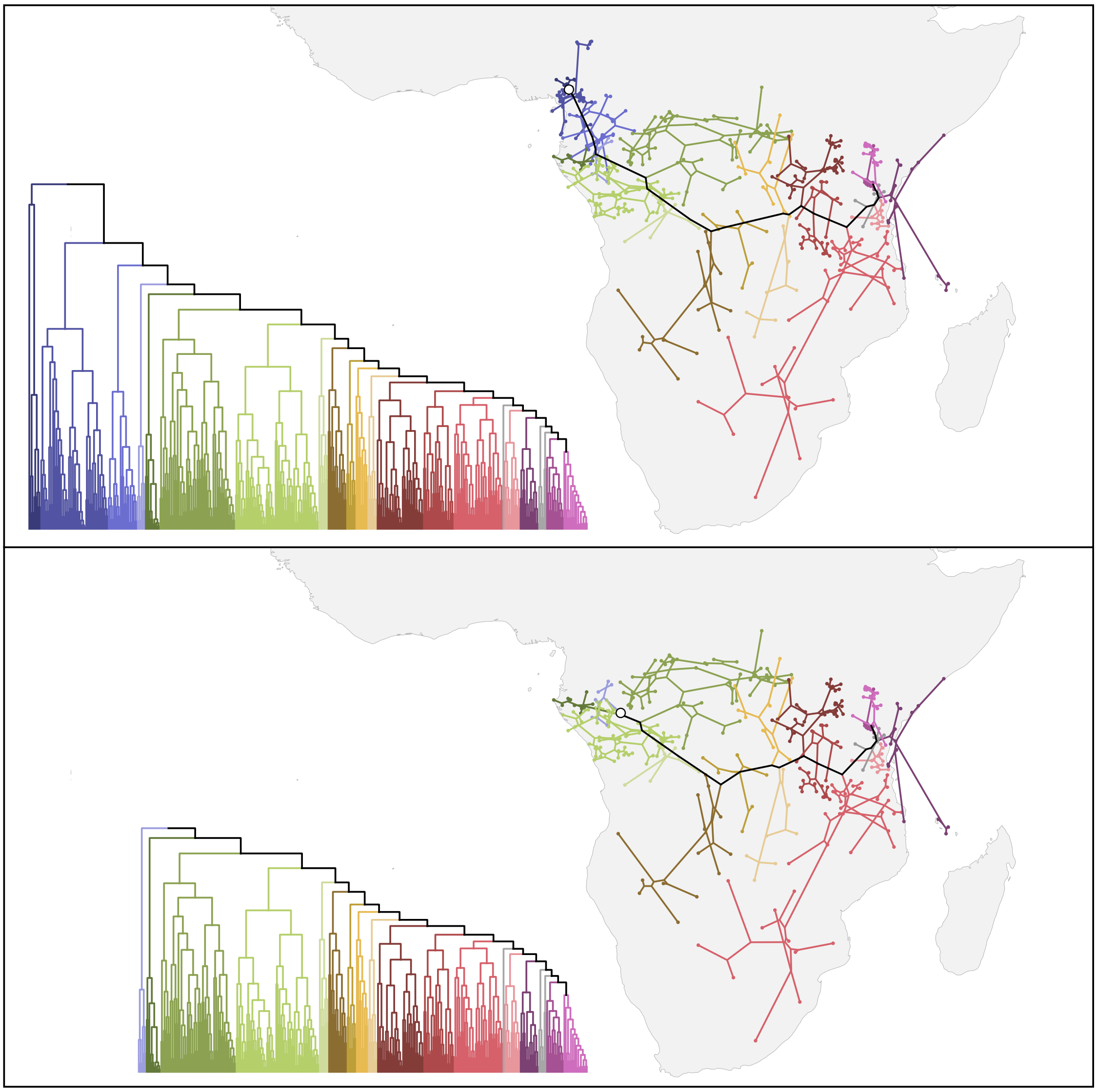

Figure S5: The effect of missing first clades.

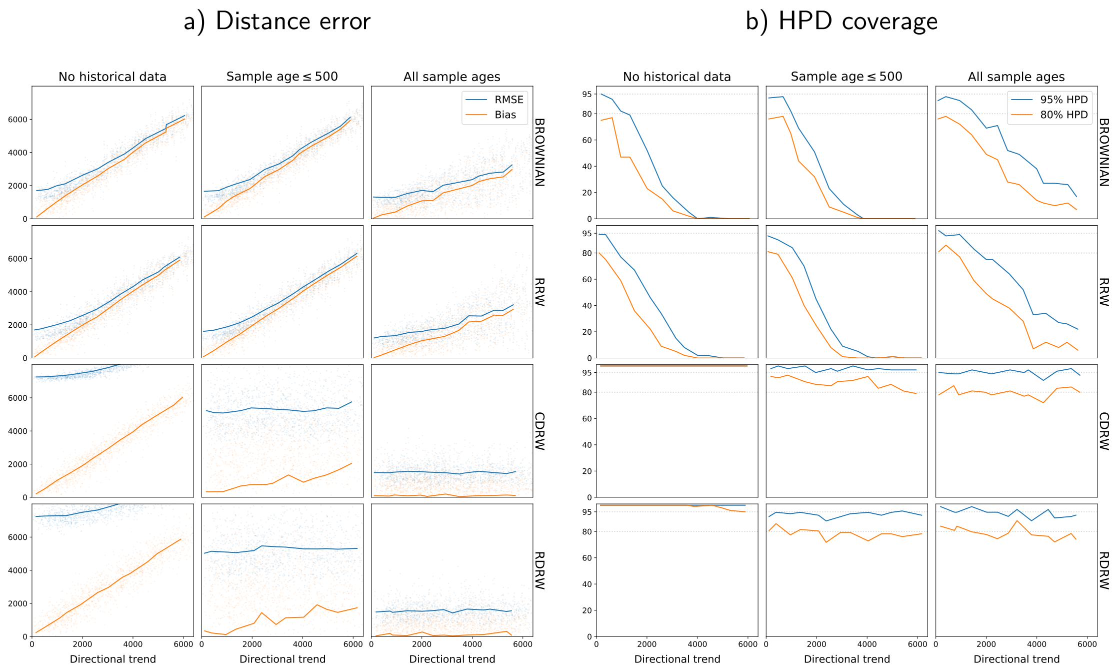

Figure S6: The performance of phylogeographic reconstructions of the root based on the MigSim simulations with varying levels of observed trend. **a)** The RMSE (blue) and the bias (orange) of the reconstruction. The dots represent single simulation runs, the lines interpolate between the average results for a specific setting for μ (trend). **b)** The empirical coverage of the 80% (blue) and 95% (orange) highest posterior density (HPD) regions.

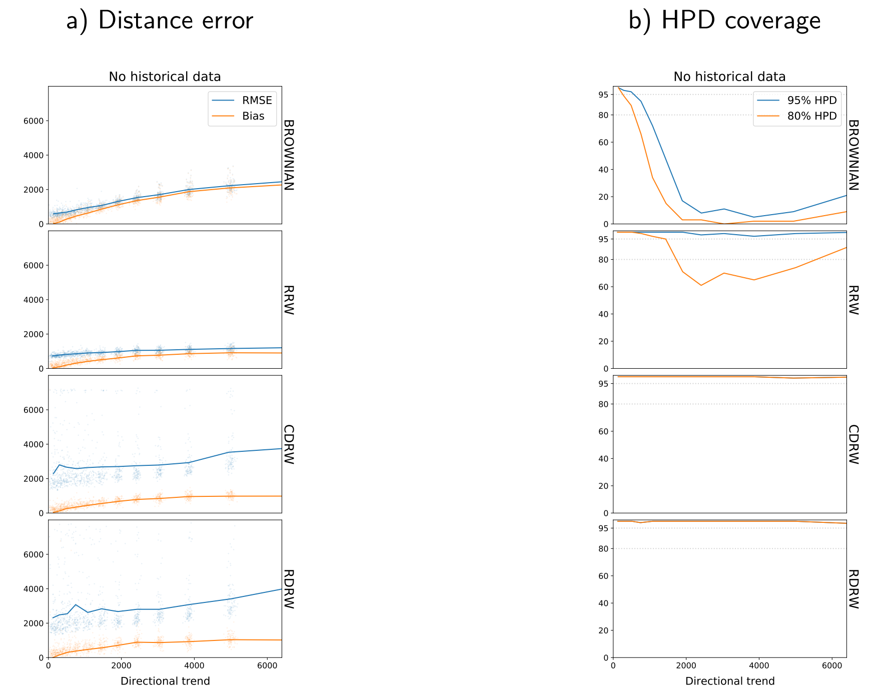

Figure S7: The performance of phylogeographic reconstructions of the root based on the ExpSim simulations with varying levels of observed trend. **a)** The RMSE (blue) and the bias (orange) of the reconstruction. The dots represent single simulation runs, the lines are averages across all runs with a specific setting for α (sector angle). **b)** The empirical coverage of the 80% (blue) and 95% (orange) highest posterior density (HPD) regions.

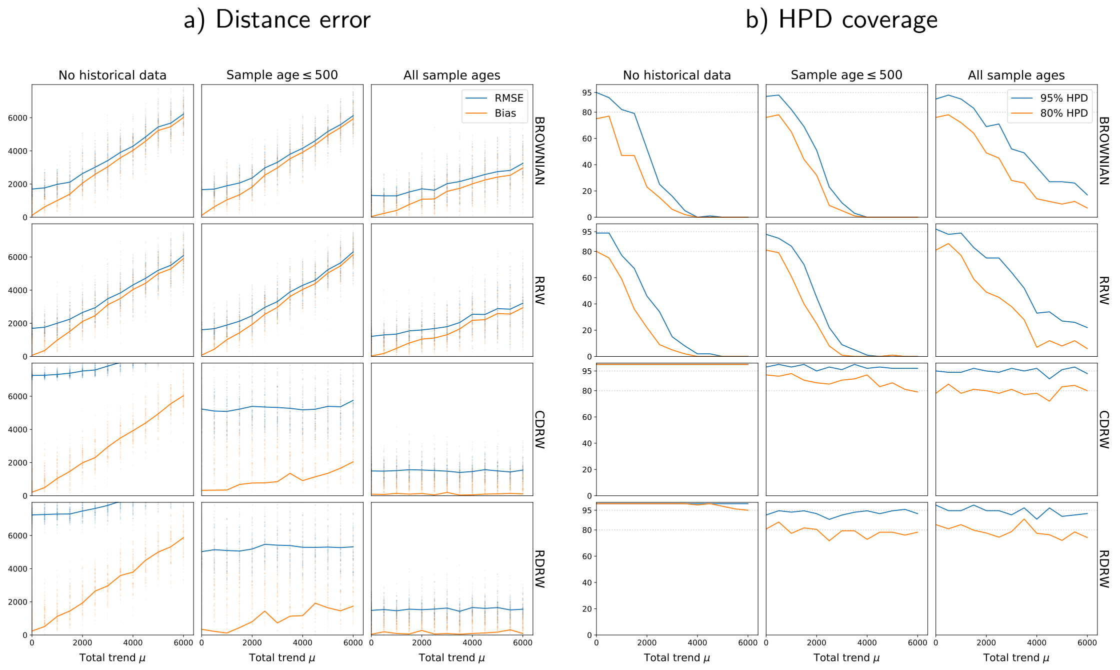

Figure S8: The performance of phylogeographic reconstructions of the root based on the MigSim simulations with varying levels of μ (total trend). **a)** The RMSE (blue) and the bias (orange) of the reconstruction. **b)** The empirical coverage of the 80% (blue) and 95% (orange) highest posterior density (HPD) regions.

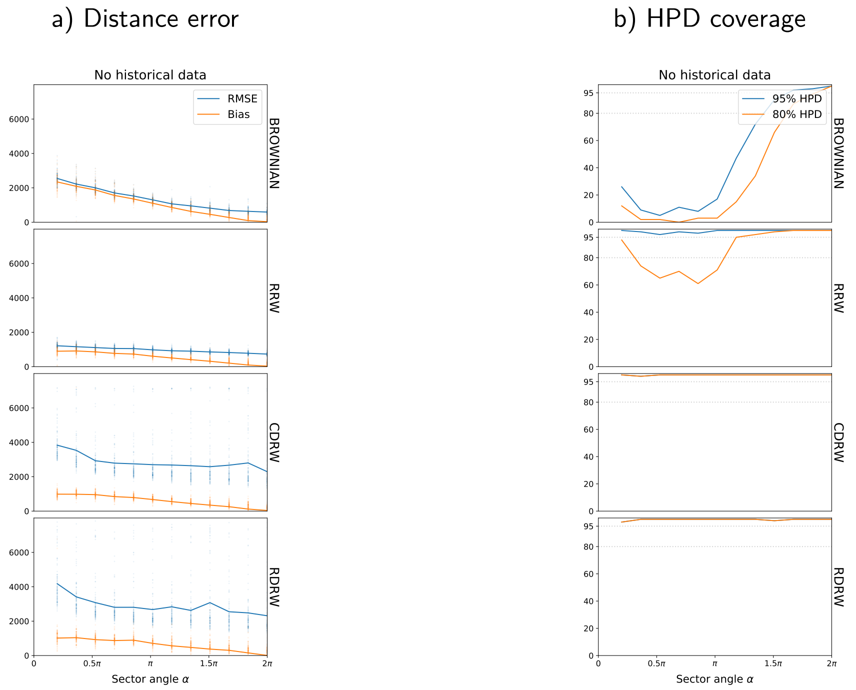

Figure S9: The performance of phylogeographic reconstructions of the root based on the ExpSim simulations with varying levels of α (sector angle). **a)** The RMSE (blue) and the bias (orange) of the reconstruction. **b)** The empirical coverage of the 80% (blue) and 95% (orange) highest posterior density (HPD) regions.

References

[1] Suchard MA, Lemey P, Baele G, Ayres DL, Drummond AJ, Rambaut A. 2018 Bayesian phylogenetic and phylodynamic data integration using BEAST 1.10. *Virus Evolution* **4**, vey016.

[2] Raup DM, Gould SJ, Schopf TJ, Simberloff DS. 1973 Stochastic models of phylogeny and the evolution of diversity. *The Journal of Geology* **81**, 525–542.

[3] Purvis A, Katzourakis A, Agapow PM. 2002 Evaluating phylogenetic tree shape: two modifications to Fusco & Cronk's method. *Journal of Theoretical Biology* **214**, 99–103.

[4] Grollemund R, Branford S, Bostoen K, Meade A, Venditti C, Pagel M. 2015 Bantu expansion shows that habitat alters the route and pace of human dispersals. *Proceedings of the National Academy of Sciences* **112**, 13296–13301.
